# The Rewarding Effect of Pictures with Positive Emotional Connotation upon Perception and Processing of Pleasant Odors—An FMRI Study

**DOI:** 10.3389/fnana.2017.00019

**Published:** 2017-03-21

**Authors:** Thomas Hummel, Therese Fark, Daniel Baum, Jonathan Warr, Cornelia B. Hummel, Valentin A. Schriever

**Affiliations:** Smell and Taste Clinic, Department of Otorhinolaryngology, Technische Universität DresdenDresden, Germany

**Keywords:** conditioning, odors, fMRI, baby pictures

## Abstract

This fMRI study was designed to investigate the effect of cross-modal conditioning in 28 female volunteers. Subjects underwent initial fMRI block design scanning during which three pleasant olfactory stimuli were presented and had to be rated with respect to intensity and pleasantness. This was followed by an odor identification task spread out over 3 days: the experimental group was rewarded for successful trials (correct odor identification) with emotionally salient photos, whilst the control group only received randomly displayed, emotionally neutral, pictures. In the final scanning session, the odors were again presented, and subjects rated pleasantness and intensity. Both pleasantness ratings and fMRI data showed effects of the rewarding procedure. Activation in nucleus accumbens and the orbitofrontal cortex confirmed the hypothesis that learnt association of odors with visual stimuli of emotionally positive valence not only increases pleasantness of the olfactory stimuli but is also reflected in the activation of brain structures relevant for hedonic and reward processing. To our knowledge, this is the first paper to report successful cross-modal conditioning of olfactory stimuli with visual clues.

## Introduction

The hedonic polarity of odors can easily be changed, especially from pleasant to unpleasant. This has been shown experimentally by combining an agreeable odor with a painful experience (e.g., Bulsing et al., 2007). The classical example for reversion of odor hedonics is food poisoning where a single exposure to an initially pleasant smell is followed by sickness is likely to produce avoidance of this smell (Bernstein, 1999). This protective function of the sense of smell is missing in anosmics who consequently experience food poisoning more frequently than normosmics (Santos et al., 2004). Changing the hedonic aspect of odors from negative to positive is also possible. A classical example is exposure to new foods which may at first smell and taste unpleasant but with experience and repeated exposure may become pleasant, e.g., the Australian spread “Vegemite®” (Kraft Foods Australia), or coriander (*Coriandrum sativum*) with its characteristic smell of the green shield bug (*Palomena prasina*) but a specific taste highly appreciated by connoisseurs. However, it appears to be easier to turn a pleasant odor into an unpleasant one than vice versa, probably due to the higher significance of an unpleasant experience (Chu, 2008; van den Bosch et al., 2015).

In order to investigate hedonic impressions elicited by olfactory stimuli, functional MRI has been used with increasing frequency during the last decade (Fulbright et al., 1998; Royet et al., 2000; Zatorre et al., 2000; Bensafi et al., 2002b; Jacob et al., 2003; Rolls et al., 2003; Konstantinidis et al., 2006; Grabenhorst et al., 2007; Knaapila et al., 2007, 2008; Lapid et al., 2008; Katata et al., 2009; Kermen et al., 2011). Olfactory-visual cross-modal phenomena may be approached by studying the effect of olfaction on visual cues, or vice versa, the effect of vision on olfactory processing, or by simultaneous presentation of both odors and images. Thus, odors have been used to modulate behavioral and electrophysiological variables in studies using ERP (Bensafi et al., 2002a; Leleu et al., 2015), MEG (Steinberg et al., 2013; Walla et al., 2003) and other methods (Seo et al., 2010; Seubert et al., 2010, 2015; Durand et al., 2013). A number of fMRI papers also report effects of odors on processing of visual cues (Gottfried et al., 2002; Gottfried and Dolan, 2004; Karunanayaka et al., 2015, 2017; Ghio et al., 2016). In contrast, little is known with regard to the effect of pictures on olfaction, even though the association with visual cues is frequently used in advertisements to emphasize the olfactory attractiveness of foods, cosmetics, cleaning, and hygiene products. Therefore, the aim of the present investigation was to study psychophysical and cerebral responses to pleasant odorous stimuli before and after associating them with pictures of varying emotional content. To approach this question, functional magnetic resonance imaging (fMRI) was chosen along with psychophysical measures. The hypothesis was that pictures with a positive emotional content (photographs of smiling infants) would have effects both on odor processing and pleasantness ratings, compared with emotionally neutral photographs of everyday objects.

## Subjects and methods

The study was performed in accordance with the “Declaration of Helsinki” (WMA, 1997), and approved by the Ethics Committee of Dresden University Medical School (reference number EK 133042012).

### Subjects

It is well-known that both olfactory function and responses to smell are related to gender (Yousem et al., 1999; Jacob et al., 2003; Royet et al., 2003; Stuck et al., 2006; Seubert et al., 2009). Thus, in order to avoid any sex-related effects, a unisex sample was investigated, with the subject's age range corresponding to the standard population in terms of normal olfactory function. Inclusion criteria required normosmia which was ascertained by a detailed interview and assessment of the sense of smell using the “Sniffin'sticks” test battery (Kobal et al., 2000; Hummel et al., 2007) in a birhinal fashion. The entire study sample comprised a total of 28 healthy, right-handed, non-smoking, female adults (19–45 years old, mean age 27 years old), who gave written informed consent to participate.

### Odorants

A set of three pleasant olfactory stimuli was used: vanillin (“VAN”), VDFLO117 with a floral scent (“FLO”), and DGFRUI067K with a fruity odor (“FRU”). In Table 1 all odors, applied dilutions and abbreviations used for convenience in this paper are listed. All odor stimuli were provided by Takasago Europe Perfumery Laboratory S.A.R.L.

**Table 1:** **Pleasant olfactory stimuli**.

**Name**	**Dilution in propylene glycol**	**Abbreviations**
Vanillin “VAN”	7 g in 30 ml	VAN
VDFLO117 “FLO”	1:2	FLO
DGFRUI067K “FRU”	1:2	FRU

### Psychophysics

Subjects were required to evaluate both intensity and pleasantness of olfactory stimuli during fMRI procedures. They were familiarized with two rating scales in a preliminary session after passing inclusion procedures. The intensity scale ran from zero (“not noticeable”) to 10 (“very strong”), whereas the hedonic scale ranged from –5 (“very unpleasant”) to 5 (“very pleasant”), with zero representing “neutral.”

Psychophysical data were analyzed using the SPSS software package (SPSS Inc., Chicago, IL, USA), applying ANOVAs for repeated measurements, and subsequent Bonferroni adjusted *t*-tests with *p* < 0.05.

### Experimental design

Subjects were pseudo-randomly assigned to two conditions, thus creating two groups of equal sizes:

- In the experimental group (XG), 12 baby photographs were used as conditioning visual stimuli with positive emotional connotation.
- In the control group (CG), 12 photographs showing neutral items of everyday life, with no emotional association, were used as visual stimuli.

The study was subdivided into three parts:

- Basic fMRI scans.
- Training/conditioning sessions: three sessions on separate days performed within 6 days.
- Final fMRI scans, 1 week after basic scans.

#### Part I—basic fMRI scans

**Figure 1:**
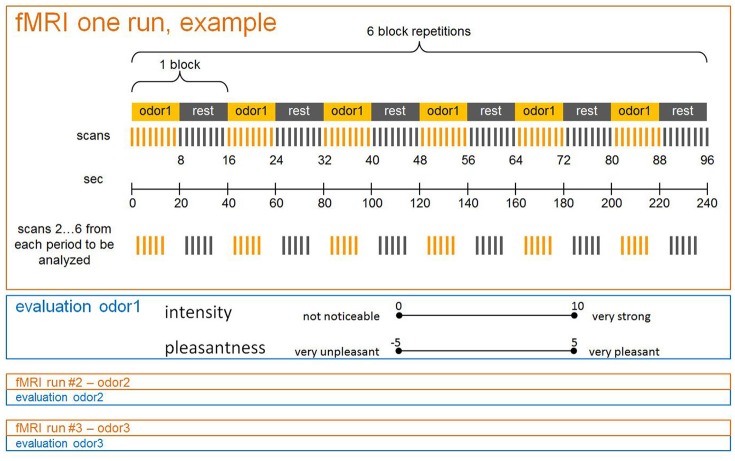
**Schematic drawing of an fMRI session including three runs (with one odor each) in a block design**. Intensity and pleasantness ratings assessed after each run.

Prior to the basic scanning procedure, subjects were successively shown all 12 pictures pertaining to their respective groups on a computer screen. During scanning, the three odors were presented in separate runs, with sequences randomized across subjects. According to the general scheme of a block design (Figure 1), each run was composed of a sequence of alternating “Off” and “On” periods, with one “Off” and one “On” period constituting one block. During “On” periods olfactory stimulation was switched on, and stopped during “Off” periods. Stimuli were presented by means of a dedicated olfactometer (Sommer et al., 2012). Pulses of odors embedded in clean air were delivered birhinally via teflon tubing (flow rate 0.75 l/min to each nostril) independent of inspiration. During “On” periods 2 s of scented air alternated with 1 s of clean air, whilst only clean air was delivered during “Off” periods. With each period comprising eight scans (20 s), and each run six repeated blocks, the duration of one run was 240 s. After each run both intensity and pleasantness of the olfactory stimuli were evaluated by means of the previously introduced rating scales. Values were orally communicated via the scanner's intercom system.

#### Part II—training/conditioning sessions

Subjects participated in three training sessions, and in the case of the experimental group, they were designed as conditioning sessions. All participants performed a computer based memory task requiring them to discriminate the three test odors from other odors not previously introduced. Odors were presented using brown glass bottles of 100 ml volume, containing 4 ml of each odor, from which subjects were asked to take sniffs. In XG, whenever a correct response occurred, subjects were rewarded by a commendation displayed on the screen (“this answer was correct”) which was followed by the display of a baby photograph. In case of failure, the item was repeated without reward, until successful identification of the target odor. Subjects had to complete 12 successful trials, thus being rewarded by the entire set of baby photographs. In CG, no rewarding displays occurred. The pictures showing neutral objects were interspersed at random during the memory task.

#### Part III—final fMRI scans

During the final scanning procedure the three odors were again presented in separate block designed runs in a randomized fashion across subjects.

**Figure 2:**
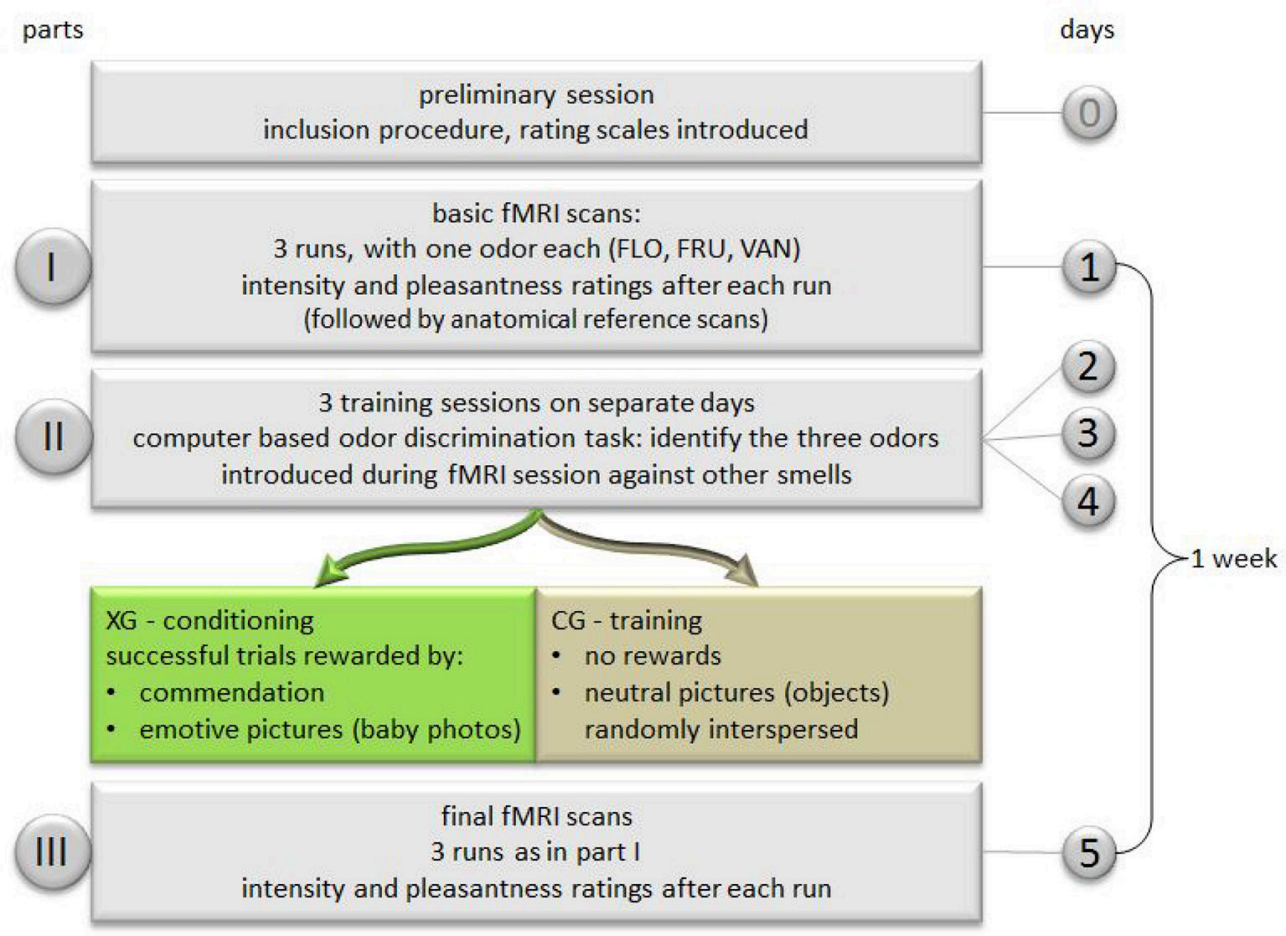
**Schematic drawing of experimental design**. Groups: XG, experimental; CG, control.

As in the basic scan, stimuli had to be rated with respect to intensity and pleasantness. A sketch of a single fMRI session is shown in Figure 1 and the entire experimental design is depicted in Figure 2.

### fMRI

Scanning was performed with a 1.5 T Sonata Scanner (Siemens, Erlangen, Germany). For functional imaging, a spin echo/echo planar imaging sequence (epfid2d1.64; ep2d.max.bold protocol) was applied using software version syngo MR 2002B 4VA21A, with echo time (*TE*) = 35 ms, repetition time (*TR*) = 2,500 ms, flip angle = 90°, and 1 average.

In part I, after functional scans, structural T1-weighted images were obtained using a magnet prepared rapid gradient echo (MP-RAGE) sequence at a resolution of 1 × 1 × 1 mm; this data set was used as reference images in fMRI analysis to account for the poor resolution of functional scans (3 × 3 × 3.7 mm).

Analysis of fMRI data was performed with the SPM8 software package (Wellcome Trust Centre for Neuroimaging, London; Friston, 2007). After spatial preprocessing (realignment, coregistration of individual functional and anatomical data sets, normalization of individual data according to the MNI standard using parameters from segmenting the structural images, and smoothing with a 6 mm full width at half maximum Gaussian kernel), scans #2 through six out of eight for each period were selected for further analysis, thus excluding scans where (a) odor flow through the tubing might not be completed, and (b) attention might be waning.

*T*-tests were first carried out to establish on-off effects of all conditions in individuals of both groups. Contrast images from this individual level of analysis were then used to test effects at the group level, where a threefold factorial design was applied to explore effects of the factors “group” (2 levels), “repetition” (2 levels), and “odor” (3 levels). Data were evaluated both in a pooled fashion, with all three odorants combined, and for the stimulants separately. Differences were tested between parts III and I (post- vs. pre-training/conditioning measurements) within both groups (XG-III vs. XG-I, and CG-III vs. CG-I), and between parts III of group XG vs. CG (XG-III vs. CG-III). Standard thresholds were set to *p*_uncorrected_ < 0.005 and 10 voxels as a minimum cluster size.

The WFU pickatlas (ANSIR Laboratory Department of Radiologic Sciences, WFU School of Medicine, Winston-Salem, NC) was used to identify activated brain regions. Regions of interest (ROI) were also established using the pickatlas. ROI_O included primary and secondary areas of olfactory processing (Gottfried, 2006): the piriform cortex, orbitofrontal cortex (OFC), the entorhinal cortex, insula, amygdala. ROI_R was set in the area of nucleus accumbens in the ventral striatum, with the intention to identify reward-related activation, and ROI_P included amygdala and orbitofrontal cortex, representing areas associated with processing of hedonics (P abbreviating pleasantness). OFC comprised bilateral supra, middle, inferior and medial orbital, and rectus gyri.

One participant from the control group had to be excluded from analysis, due to a set of corrupted MRI data.

## Results

### Psychophysics

#### Intensity

Intensity ratings revealed significant differences among odors: In the analysis of ratings at baseline from the entire sample [*F*_(2, 52)_ = 34.0], VAN was significantly weaker than the other two odors. FLO received the highest scores, and was significantly stronger than VAN and FRU. However, in terms of odor intensity at baseline, there was no significant difference between groups [*F*_(1, 26)_ = 1.84, *p* = 0.19].

In both the experimental and control groups, differences in odor intensity did not change after association of the odors with baby photos or emotionally neutral pictures respectively [controls: *F*_(1, 13)_ = 0.20, *p* = 0.66; baby photo group: *F*_(1, 13)_ = 0.13, *p* = 0.73].

#### Pleasantness

Most individual pleasantness ratings were above zero, although none of the three odors was unanimously rated as pleasant. At baseline, there was no significant difference between the two groups in terms of odor pleasantness [*F*_(1, 26)_ = 0.96, *p* = 0.34]; in addition, no significant difference was found between the three odors [*F*_(2, 52)_ = 2.05, *p* = 0.14].

**Figure 3:**
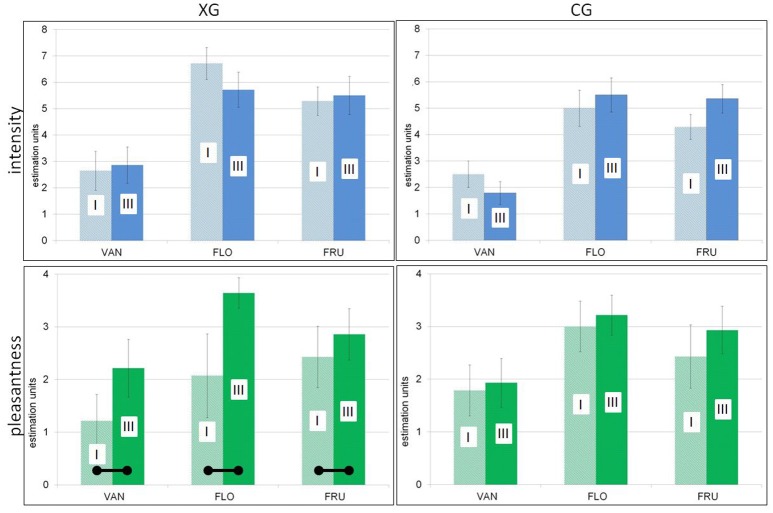
**Psychophysics (means, SEM), comparisons between parts I (initial session), and III (after training/conditioning). (Top)**, Intensity ratings. **(Bottom)**, Pleasantness ratings. **(Left)**, Experimental group. **(Right)**, Control group. – Significant increases of pleasantness in the experimental group after conditioning depicted by horizontal bars.

A significant effect of the conditioning procedure could be established in the experimental group: pleasantness ratings of all three odors were found to be significantly increased after as compared to before the conditioning session [*F*_(1, 13)_ = 5.50; *p* = 0.036]. In contrast, for CG, although olfactory stimuli were rated more pleasant in part III than prior to training, these increases were not significant [*F*_(1, 13)_ = 0.14; *p* = 0.87]. The findings for both groups are demonstrated in Figure 3. Pleasantness and intensity ratings were uncorrelated for FRU and FLO; however, a significant positive correlation was found in case of VAN (entire sample, *r* = 0.73; *p* < 0.05).

Both intensity and pleasantness ratings are listed in Table 2.

**Table 2:** **Means and standard deviations of psychophysical data**.

**Group**		**VAN**	**FLO**	**FRU**
XG	INT I	2.64 ± 2.76	6.71 ± 2.27	5.29 ± 2.02
	INT III	2.86 ± 2.57	5.71 ± 2.49	5.50 ± 2.71
	HED I	1.21 ± 1.89	2.07 ± 2.97	2.43 ± 2.17
	HED III	2.21 ± 2.04	3.64 ± 1.08	2.86 ± 1.83
CG	INT I	2.50 ± 1.87	5.00 ± 2.54	4.29 ± 1.77
	INT III	1.79 ± 1.63	5.50 ± 2.41	5.36 ± 2.02
	HED I	1.79 ± 1.81	3.00 ± 1.80	2.43 ± 2.24
	HED III	1.93 ± 1.73	3.21 ± 1.42	2.93 ± 1.69
All	INT I	2.57 ± 2.32	5.86 ± 2.52	4.79 ± 1.93
	INT III	2.32 ± 2.18	5.61 ± 2.41	5.43 ± 2.35
	HED I	1.50 ± 1.84	2.54 ± 2.46	2.43 ± 2.17
	HED III	2.07 ± 1.86	3.43 ± 1.26	2.89 ± 1.73

*INT, Intensity; HED, Hedonics (Pleasantness); XG, Experimental group; CG, control group; All, entire sample; I, basic session (before training/conditioning); III, final session (after training/conditioning). VAN, FLO, FRU: odors*.

### fMRI

**Figure 4:**
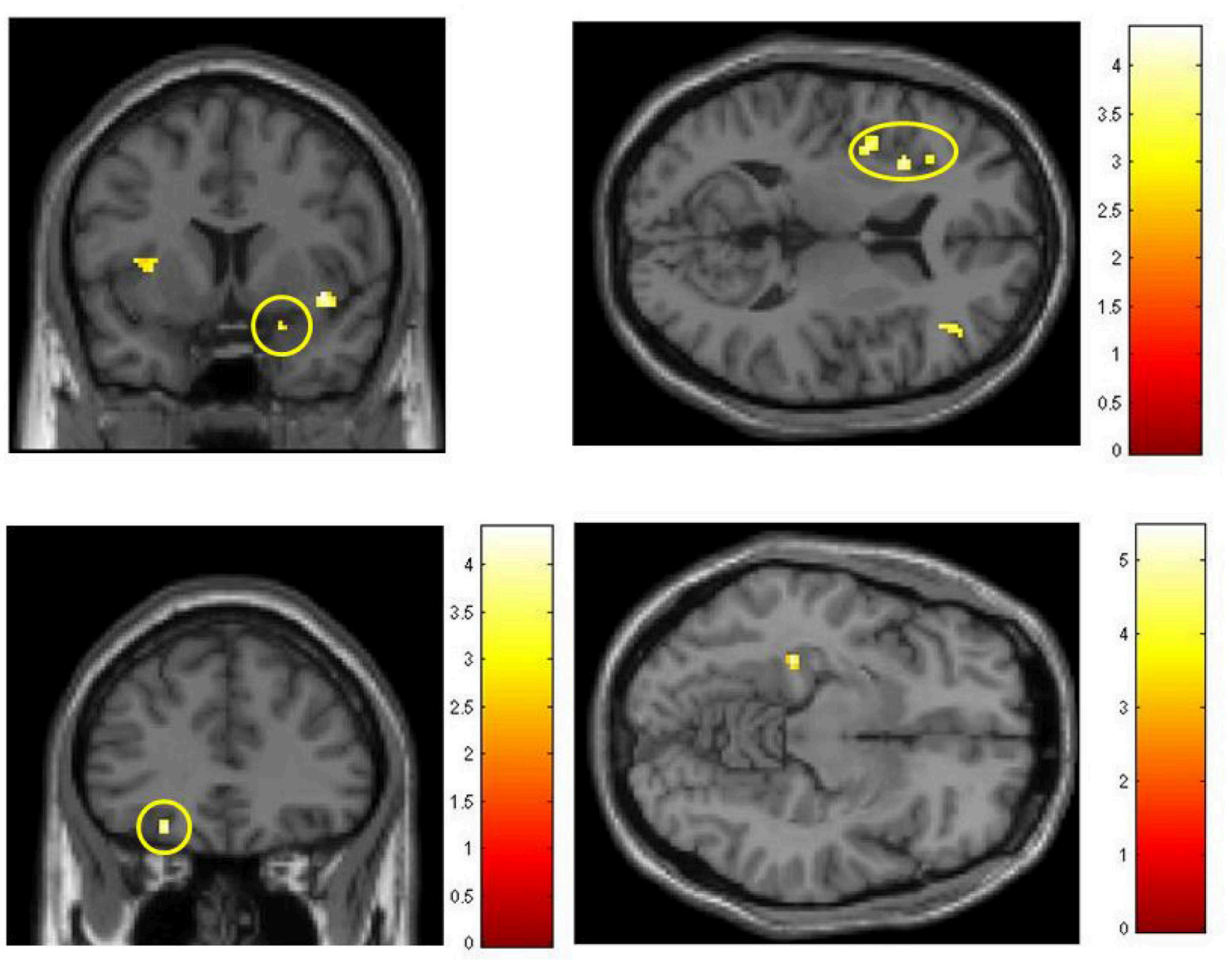
**Olfactory activations from part I (initial session), obtained with ROI-O (areas associated with olfactory processing); colored scales indicate t values. Top left:** Stimulant FLO, activation in the piriform cortex. **Top right:** Stimulant FLO, activation in the insula. **Bottom left:** Stimulant FRU, OFC activation. **Bottom right:** Stimulant VAN, parahippocampal activation.

Exposure to FLO, FRU, and VAN odors induced activation in brain regions associated with olfactory processing. In Figure 4, examples from the initial part I of the study demonstrate BOLD effects in the piriform and orbitofrontal cortices, insula and parahippocampal cortex, as obtained with ROI_O. Activation of the piriform cortex was only revealed by setting the initial error probability to *p* < 0.01 prior to ROI analysis.

With respect to the effect of conditioning with emotionally positive photos, pooled analysis of all three odors was first performed. Comparisons were calculated between groups for part III, and between parts III and I for the experimental group. Neither analysis with ROI_P, to check for activations in regions involved in pleasantness processing, nor with ROI_R, testing reward relevant activation, yielded significant results in the pooled approach.

**Figure 5:**
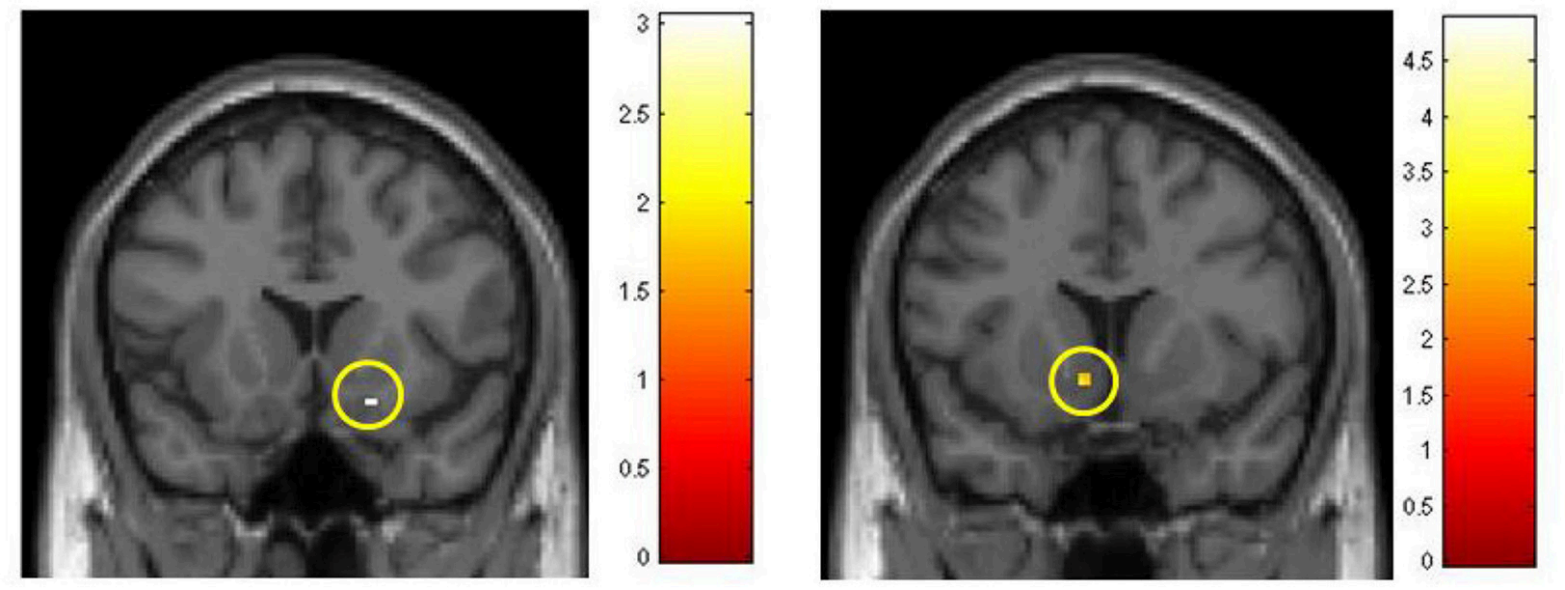
**Activations in the region of nucleus accumbens, obtained with ROI-R**. Colored scales represent *t*-values. **Left:** Comparison of FRU effects, part III vs. part I in the experimental group (post vs. pre-conditioning with emotionally salient photos). **Right:** Comparisons of VAN effects between groups in part III, after training/conditioning sessions.

Selective analyses with the three separate odors were then performed in the same fashion (Table 3). Using this approach significant results were obtained. In the contrast XG-III vs. CG-III, ROI analysis using ROI-P revealed OFC activation for all three odors. In addition, with VAN stimulation the area of nucleus accumbens showed activation with the ROI_R analysis. In the comparison between parts III and I for XG with the stimulant FRU a similar activation was revealed applying ROI-R analysis. Figure 5 summarizes these findings.

**Table 3:** **Significant activations**.

	**Odor**		***T***	***p*(unc)**	***x***	***y***	***z***	**Side**	**Area**
1-sample *t*-tests, part I all subjects	FLO		4.38	0.00013	–30	16	10	L	Insula
			4.33	0.00015	40	8	–8	R	Insula
			4.08	0.00027	46	44	–12	R	OFC
			3.98	0.00034	–38	2	8	L	Insula
			3.85	0.00047	–30	56	14	L	OFC
			3.49	0.00109	–32	26	6	L	Insula
			3.36	0.00100	22	8	–20	R	piriform cortex
	FRU		4.20	0.00020	–24	34	–16	L	OFC
	VAN		5.45	0.00001	–30	–32	–10	L	parahipp. Gyrus
			4.25	0.00018	–24	52	4	L	OFC
			3.96	0.00035	–28	–36	2	L	hippocampus
			3.80	0.00053	36	54	10	R	OFC
			3.61	0.00082	40	50	18	R	OFC
		**Contrast**	***T***	***p*****(unc)**	***x***	***y***	***z***	**Side**	**ROI**
3-factorial analysis	FLO	XG-III vs. CG-III	3.33	0.0006	–32	38	–4	L	OFC
	FRU	XG-III vs. CG-III	2.98	0.0017	–8	28	–12	L	OFC
			2.81	0.0029	–12	34	–10	L	
			2.71	0.0039	–12	38	–8	L	
	VAN	XG-III vs. CG-III	3.20	0.0009	26	32	–12	R	OFC
			2.66	0.0044	22	30	–14	R	
			3.19	0.0009	–8	12	–8	L	accumb.ncl.
	FRU	XG-III vs. XG-I	3.04	0.0014	18	16	–14	R	accumb.ncl.

*T, t-value; P(unc), uncorrected p; x, y, z, MNI coordinates; 1-sample t-tests with ROI-O*.

## Discussion

The current study revealed that the pleasantness of odors increased when their correct recognition was associated with photos of happy babies whereas no such effect occurred when the same odors were presented in a random fashion with emotionally neutral pictures.

Several fMRI papers have shown effects of odors on various visual variables. Gottfried and coworkers (Gottfried et al., 2002; Gottfried and Dolan, 2004) performed conditioning/learning experiments where odors affected processing of visual stimuli dependent on the different hedonic values of the odors. Similarly, Karunanayaka et al. (2015) found that visual cues produced activations in different areas after pairing them with a lavender smell of varying intensities or presenting them unpaired. In another study, Karunanayaka et al. (2017), again using both paired visual-olfactory and unpaired visual stimuli, observed Default Mode Network (DMN) deactivation only in the odor-visual association paradigm. Ghio et al. (2016) reported an effect of associating odors with novel objects vs. non-associated objects in terms of different activations. In a different approach, Villemure et al. (2006, 2012) investigated the soothing effect of pleasant odors on pain processing.

By contrast reports on influencing olfactory sensory response via psychophysical or behavioral variables are scarce. Taste has been found to affect olfaction: Barkat et al. (2008) reported an increase in odor pleasantness as an effect of the association with sweet taste, and Yeomans and Prescott (2016) assessed several parameters including odor liking and found they were altered after pairing odors with sweet or bitter tastes. To our knowledge only one publication addressed the question of vision affecting olfaction: Koza et al. (2005) found that colors intensified orthonasal as opposed to retronasal olfactory perception. However, this paper is exclusively focused on psychophysical parameters.

Thus it appears that the present study is the first to establish evidence of emotional visual stimuli enhancing pleasantness of odors with respect to both psychophysical and brain imaging data.

An important aspect for the interpretation of the results presented here is the constancy of odor intensity. There were no significant differences of intensity ratings between the groups at baseline (part I), or in part III, and none between parts I and III for either group. This supports the idea that the significant increase of pleasantness ratings found in the experimental group in part III is independent of intensity and specifically reflected to the hedonic aspect. Moreover this also applies to the imaging results where significant differences were observed when contrasting XG vs. CG in part III, and parts III vs. I for the experimental group. Apparently the rewarding experience of the conditioning sessions brought about increased pleasantness ratings for the olfactory stimuli and associated activation in OFC and nucleus accumbens. Thus the results support the hypothesis that visual cues with a positive emotional connotation enhance the basic agreeableness of fragrances and induce activation of cortical networks related to hedonics and reward (Gottfried et al., 2002; Gottfried and Dolan, 2003; Villemure et al., 2012; Jiang et al., 2015; Rolls, 2015).

However, certain drawbacks concerning the results need to be discussed. Activation of the basic areas of olfactory processing would have been expected to occur for all three odors, but were only observed partially for any of the odor conditions. Furthermore, group comparisons yielding the activations in OFC and nucleus accumbens did not occur for all single odor nor for all pooled comparisons—in spite of a lenient, uncorrected error threshold. These shortcomings could perhaps be explained by low odor concentrations barely supra-threshold intensities for some odor-subject pairs or poor signal quality with a 1.5 T scanner. However, as Lieberman and Cunningham (2009) have pointed out, uncorrected *p*-values should not be rejected out of hand, as they may reveal weaker effects, and can be compensated by repetitive investigations and meta-analyses which will ultimately reveal “the truth.” In this case, further studies, for example with stronger odor concentrations and/or superior data acquisition, may contribute to this aim.

## Ethics statement

The study protocol was approved by the Ethics Committee of the Medical Faculty of the TU Dresden, Dresden, Germany Verbal, and written consent was obtained after participants received detailed information (both verbal and written) about the experiment.

## Author contributions

TH, JW, CH, DB, and TF developed the idea and the design of the study; TF and DB ran the experiments; CH, TF, DB, and TH were involved in data analysis; TH, CH, TF, DB, JW, and VS contributed to the writeup.

## Funding

This research was supported by Takasago Inc., Paris, France.

### Conflict of interest statement

The authors declare that the research was conducted in the absence of any commercial or financial relationships that could be construed as a potential conflict of interest. The reviewer VB and handling Editor declared their shared affiliation, and the handling Editor states that the process nevertheless met the standards of a fair and objective review.
